# The Challenge of Diagnosing Invasive Pulmonary Aspergillosis in Children: A Review of Existing and Emerging Tools

**DOI:** 10.1007/s11046-023-00714-4

**Published:** 2023-04-08

**Authors:** Daniel K. Yeoh, Brendan J. McMullan, Julia E. Clark, Monica A. Slavin, Gabrielle M. Haeusler, Christopher C. Blyth

**Affiliations:** 1 Department of Infectious Diseases, Perth Children’s Hospital, 15 Hospital Avenue, Perth, WA 6009 Australia; 2 Sir Peter MacCallum Department of Oncology, University of Melbourne, Parkville, VIC Australia; 3 National Centre for Infections in Cancer, Peter MacCallum Cancer Centre, Melbourne, VIC Australia; 4 Murdoch Children’s Research Institute, Parkville, VIC Australia; 5 Wesfarmers Centre of Vaccines and Infectious Diseases, Telethon Kids Institute, University of Western Australia, Perth, WA Australia; 6 Department of Immunology and Infectious Diseases, Sydney Children’s Hospital, Randwick, NSW Australia; 7 School of Women’s and Children’s Health, UNSW, Sydney, NSW Australia; 8 Infection Management Service, Queensland Children’s Hospital, Brisbane, QLD Australia; 9 School of Clinical Medicine, Children’s Health Queensland Clinical Unit, The University of Queensland, Brisbane, QLD Australia; 10 Department of Infectious Diseases, Peter MacCallum Cancer Centre, Melbourne, VIC Australia; 11 Department of Infectious Diseases, Royal Children’s Hospital, Parkville, VIC Australia; 12 The Paediatric Integrated Cancer Service, Melbourne, VIC Australia; 13 Department of Microbiology, PathWest Laboratory Medicine WA, Nedlands, WA Australia; 14 School of Medicine, University of Western Australia, Perth, WA Australia

**Keywords:** Invasive pulmonary aspergillosis, Aspergillus, Diagnostic imaging, Diagnostic mycology, Children

## Abstract

Invasive pulmonary aspergillosis remains a major cause of morbidity and mortality for immunocompromised children, particularly for patients with acute leukaemia and those undergoing haematopoietic stem cell transplantation. Timely diagnosis, using a combination of computed tomography (CT) imaging and microbiological testing, is key to improve prognosis, yet there are inherent challenges in this process. For CT imaging, changes in children are generally less specific than those reported in adults and recent data are limited. Respiratory sampling by either bronchoalveolar lavage or lung biopsy is recommended but is not always feasible in children, and serum biomarkers, including galactomannan, have important limitations. In this review we summarise the current paediatric data on available diagnostic tests for IPA and highlight key emerging diagnostic modalities with potential for future use.

**Supplementary Information:**

The online version contains supplementary material available at 10.1007/s11046-023-00714-4.

## Introduction

Invasive pulmonary aspergillosis (IPA) remains a significant cause of morbidity and mortality in immunocompromised children [1]. The lung is the most frequent primary site of *Aspergillus* spp. infection in immunocompromised children with infection occurring following inhalation of ubiquitous airborne conidia. In the setting of impaired innate and adaptive immunity, germination is followed by angioinvasion, necrosis and haematogenous dissemination [2]. *Aspergillus fumigatus* is the most frequently isolated species in children, followed by *A. flavus, A niger* and *A. terreus* [1]*.* Mortality rates of up to 50% have been reported in severely immunocompromised children [3].

Children at highest risk of IPA include those undergoing haematopoietic stem cell transplantation (HSCT) or receiving intensive chemotherapy for haematological malignancy, particularly children with acute myeloid leukaemia (AML), high-risk acute lymphoblastic leukaemia (ALL) or relapsed leukaemia [1, 4]. Prolonged neutropenia, high-dose steroid exposure, graft versus host disease and more recently identified, increasing age are specific risk factors for IFD in this context [5]. Paediatric solid organ (particularly heart and lung) transplant recipients are also at risk of IPA [1, 3], as are children with inborn errors of immunity, specifically chronic granulomatous disease (CGD), hyper immunoglobulin (Ig) E syndrome (in the context of underlying structural lung disease) and severe congenital neutropenia [6, 7]. Severe aplastic anaemia is also a risk factor for IPA [8], with children progressing to HSCT at highest risk [5].

Early diagnosis is key to facilitate timely initiation of optimal antifungal therapy, yet this can be challenging as clinical features are non-specific, with most patients presenting with isolated fever, occasionally accompanied by chest pain, cough or dyspnoea [9–11]. The diagnosis of IPA is based on a combination of diagnostic imaging, microbiological testing of respiratory samples and serum biomarkers. For children, there are important considerations in diagnostic testing, including differences in imaging manifestations and laboratory test performance. In this review we summarise available literature assessing current radiological and microbiological techniques in the diagnosis of IPA in children and identify emerging approaches which may improve IPA diagnosis in the future.

## Diagnostic Imaging in IPA

Computed Tomography (CT) is the diagnostic imaging modality of choice for children with suspected IPA [12–18]. Compared to plain radiograph, CT is more sensitive and can detect subtle findings earlier [14, 15, 19]. The European Organization for Research and Treatment of Cancer/Invasive Fungal Infections Cooperative Group (EORTC/MSG) include specific CT imaging changes in their definition of IPA, based predominantly on adult studies. These include dense, well-circumscribed nodules with or without a halo sign, the air crescent sign, cavities, and wedge-shaped and segmental or lobar consolidation [15, 20]. Notably these criteria were designed primarily to improve quality of research studies rather than for clinical practice and, more recently, a broader range of imaging findings than previously appreciated has been noted in adult cases of IPA [14, 15]. Importantly, there are key differences in imaging changes in children with IPA compared with adults [14, 21, 22].

### CT Imaging in Paediatric IPA: Typical Findings

**Fig. 1:**
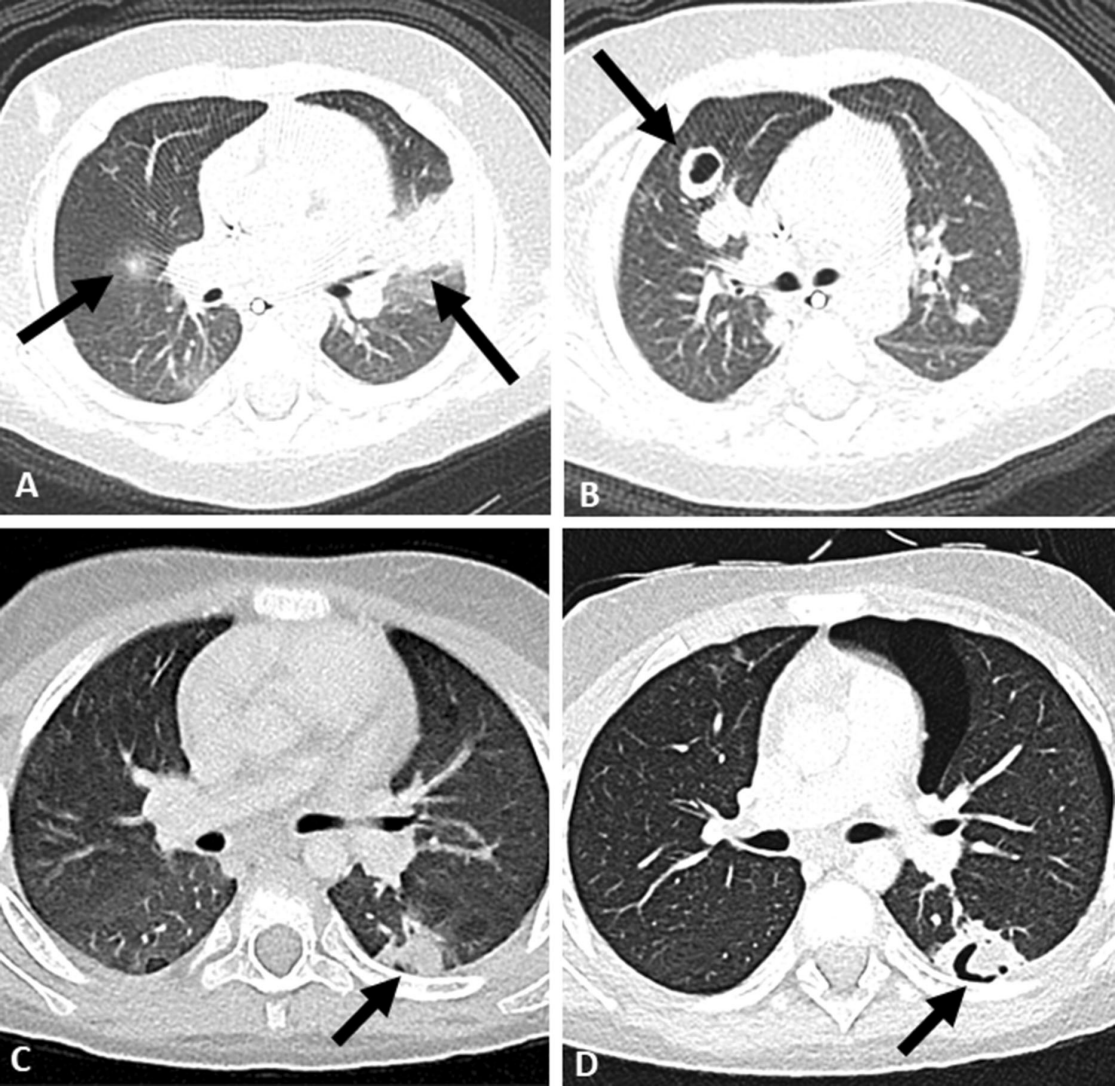
Imaging findings in invasive pulmonary aspergillosis: **A**, **B**—6 month old with acute lymphoblastic leukaemia complicated by *Aspergillus fumigatus* infection showing **A** left upper lobe wedge shaped/segmental consolidated and right upper lobe nodule with surrounding ground glass halo and **B** right upper lobe nodule with central cavitation; **C**, **D**—8 year old with acute lymphoblastic leukaemia complicated by disseminated *Aspergillus flavus* infection showing **C** left lower lobe nodule at diagnosis with **D** evolution to air crescent sign on follow up imaging

Several studies have documented the CT imaging findings of children with IPA (Table 1) [3, 9, 10, 23–25], predominantly in children with malignancy or undergoing HSCT. Most studies included a small number of patients, with one exception which described the CT and or plain radiograph findings in 110 children with IPA [3]. Similar to adults, nodular opacities are most frequently described (59–100%), followed by wedge shaped/segmental or lobar consolidation (21–63%). Cavitation (0–43%) and the air crescent sign (0–21%) are less frequently seen (Fig. 1). The prevalence of the halo sign is variable ranging from 0 to 100% in individual series.

**Table 1:** Findings on computed tomography (CT) imaging of invasive pulmonary Aspergillosis from previous paediatric studies

Author (Country)	Year of publication (study range)	CT scans of IPA	Risk factors	Radiological findings					
				Nodules/mass	Halo	Cavity	Air crescent	Consolidation (Wedge shaped or lobar)	Other
Taccone [10] (Italy)	1993 (1987–1991)	14^%^	Malignancy/HSCT	13/14 (93%)	2/14 (14%)	6/14 (43%)	3/14 (21%)	3/14 (21%)	Effusion (1)
Thomas [23] (UK)	2003 (10-year retrospective)	8^#^	Malignancy/PID (SCID/CGD)/Vasculitis	6/8 (75%)	0/8	2/8 (25%)	0/8	5/8 (63%)	Effusion (3)
Gasparetto [24]	2008 (1993–2006)	9^^^^	HSCT	6/9 (67%)	5/9 (56%)	0/9 (0%)	0/9 (0%)	5/9 (56%)	Effusion (1) Ground Glass (1) Tree in bud (2)
Burgos [3] (US)	2008 (2000–2005)	110^*^^#^	Malignancy/HSCT/PID (SCID/CGD)/SOT	65/110 (59%)	12/110 (11%)	27/110 (25%)	3/110 (3%)	2/110 (2%) (wedge-shaped)	“Other infiltrate” (39)
Han [25] (Korea)	2015 (2009–2013)	37^#^ (35 prob, 2 prov)	Malignancy/HSCT/Aplastic Anaemia	N/A	29/37 (78%)	2/37 (5%)	0/37	8/37 (22%)	Nil
Bain [9] (Brazil)	2022 (2008–2014)	5^#^	Malignancy	5/5 (100%)	5/5 (100%)	2/5 (40%)	0/5	2/5 (40%)	Effusion (1) Ground glass (3)

CGD, Chronic Granulomatous Disease; HSCT, Haematopoeitic Stem Cell Transplantation; IPA, Invasive Pulmonary Aspergillosis; PID, Primary Immunodeficiency; SCID, Severe Combined Immunodeficiency; SOT, Solid Organ Transplantation

^%^“documented IPA” based on nasal culture, sputum, serum, bronchoalveolar lavage or surgical/autopsy findings”

*CT and plain radiograph findings combined, included patients with “other infiltrates” & signs of lung infection as well

^#^Probable/proven based on European Organization for Research and Treatment of Cancer Mycoses Study Group (EORTC/MSG) Criteria [15, 20]

^^^^diagnosis based on culture and histological evidence of tissue invasion

Nodular lesions are consistently the most frequent CT finding in pulmonary IPA, as such their presence, in an immunocompromised child with a consistent clinical picture, should prompt further investigation and consideration of pre-emptive antifungal therapy. Notably, however, nodules have a wide differential diagnosis in immunocompromised patients including other infectious and non-infectious processes, therefore microbiological confirmation remains important [26–28]. In previous small paediatric studies, larger nodules or mass like opacity appear more suggestive of fungal infection, although these also occurred in bacterial and viral infection [24, 29].

Another important early finding in IPA in neutropenic hosts is the halo sign, present in > 70% of adult cases at baseline, although with progression of disease prevalence decreases rapidly over time [14, 30]. Like nodular opacities, the halo sign has a broad differential and can be seen with bacterial and viral infection as well as non-infective processes [21, 22]. A recent systematic review reported high specificity of the halo sign (> 90%) for diagnosis of invasive fungal disease (IFD) in immunocompromised adults and children [31] although data on specificity for IPA are conflicting [14, 27]. Notably, although earlier paediatric studies report a lower frequency of the halo sign (0–11%) [3, 10, 23], prevalence is higher in more recent studies (56–100%), possibly reflecting improvements in CT resolution and earlier timing of diagnostic scans [9, 25]. In two previous paediatric studies, presence of the halo sign was highly specific for pulmonary IFD, although very few patients with IPA were included [24, 29]. More recently, Han et al. found that although more frequent in children with proven/probable IPA, the halo sign occurs commonly in children without IPA (78% vs. 41%) [25].

Cavitation and the air crescent sign appear in later stages of infection in neutropenic adults, occurring concurrently with bone marrow recovery [14, 30]. Similarly, cavitation of lesions during marrow recovery has been documented in children with IPA on plain radiographs [32]. Amongst paediatric CT studies, the analysis by Taccone et al. which reported the highest prevalence of cavitation (43%) included findings on follow up CT studies, noting progression to cavitation in a number of cases 2–3 weeks after treatment, coinciding with neutrophil recovery [10]. In paediatric studies including initial diagnostic CT only, cavitation occurs in less than a quarter of cases and the air crescent sign is infrequent (0–3%) (Table 1). Notably, prevalence of cavitation did not significantly differ between age groups in the largest paediatric series to date [3]. Considering the low prevalence in children and overall low specificity of these signs [14, 15], the utility of the air crescent sign and cavitation as individual findings is limited in children with suspected IPA.

A range of other findings including tree-in-bud opacity, ground glass opacity and effusion have been identified in paediatric IPA (Table 1) similar to recent adult data [14]. As these changes are not specific for IPA, their diagnostic utility in the absence of more typical findings of IPA is less certain. In practice, for children at high risk of IPA, any new area of change on CT in the setting of prolonged fever should prompt consideration of further microbiological investigation (see below) [16]. Where microbiological testing is inconclusive, re-imaging to assess evolution of changes after an interval of close clinical monitoring with or without pre-emptive antifungal therapy may be a reasonable approach in this setting (Supplementary Fig. 1).

### CT Imaging: Unanswered Questions

Paediatric studies documenting imaging changes in IPA predominantly include patients and images from the 1990s or early 2000s, including the largest series by Burgos et al. [3, 10, 23, 24] More recent studies have included relatively small numbers (< 40) [9, 25]. Updated studies documenting CT findings in children with IPA in the setting of improvements in CT techniques, changes in immunosuppressive regimens and supportive care including use of anti-mould prophylaxis are required [14, 22]. Furthermore, important differences in imaging findings between adult risk groups have been observed^4^ yet there are limited data on the CT in IPA for paediatric solid organ transplant recipients and children with primary immunodeficiency specifically [6]. Similarly, adult studies have documented differences in imaging findings between IPA and pulmonary mucormycosis [33, 34], equivalent paediatric data are lacking.

CT imaging changes are a key factor in clinical decision making in patient with suspected IPA, yet reliability of radiology interpretation of scans has not been thoroughly assessed. For example, identification of nodules can vary substantially between radiologists assessing adult CT scans [35, 36]. Similarly, agreement in radiologist assessment of the halo sign is imperfect [22]. In contrast, Bain et al. reported substantial agreement between two radiologists in identifying nodules (Kappa = 0.649), halo sign (Kappa = 0.609) and cavities (Kappa = 0.860) from CT images of 40 children diagnosed IFD (29 possible), although few cases of IPA (5) were included [9]. As typical imaging changes are not consistently present in IPA in children, and common findings (e.g. nodules, consolidation) have a broad differential, overall radiologist impression of the likelihood of IPA (potentially influenced by additional factors including clinical information provided on the request for imaging, risk factors and antifungal use) is likely to influence treatment decisions, particularly where confirmatory microbiological sampling is unavailable or inconclusive. One paediatric study, from 1997, of 48 children with pulmonary complications of haematological malignancy found overall radiologist impression of CT findings had good diagnostic accuracy (area under ROC curve = 0.78) for fungal pneumonia (11 cases; including 7 IPA) [29]; although there are no recent similar studies. Ideally treatment decisions in this context should be discussed in multi-disciplinary setting with radiologists, oncologists and infectious diseases specialists considering all clinical, imaging and microbiological information available.

### Emerging Imaging Modalities

Several existing imaging technologies, including CT pulmonary angiography (CTPA) and magnetic resonance imaging (MRI) have potential advantages in diagnosis of IPA, although paediatric data remain limited. In adults, CTPA can detect early vessel occlusion secondary to angioinvasion, a hallmark of pulmonary IFD [37]. In a study of 100 adult haematology patients with prolonged fever and macronodular pulmonary infiltrate on initial screening CT, vessel occlusion on CTPA had high sensitivity (100%) for pulmonary IFD, allowing earlier cessation of antifungal therapy in patients with negative scans [37]. Thus far CTPA has not been assessed in diagnosis of IPA in children [13, 38]. With the absence of radiation exposure in MRI, this modality is attractive in children, however data for its diagnostic use in IPA is limited [14]. Furthermore, there are inherent limitations with MRI including movement artefact, poorer resolution of lung images using standard sequences and the need for patient co-operation for prolonged periods which may be particularly difficult for paediatric patients [14].

Fluorodeoxyglucose-positron emission tomography (FDG-PET) imaging is a promising emerging modality for the diagnosis of IFD in immunocompromised patients. Traditionally used to stage malignancies, FDG is also taken up by activated phagocytes, and therefore can detect areas of focal inflammation and infection [39]. In adult cancer patients, FDG-PET CT may be superior to conventional imaging in identifying occult infective foci and dissemination during initial diagnostic workup of IFD, including IPA [40, 41]. FDG-PET CT has also shown promise in the diagnosis and staging of infection in children in a small number of studies, with few (< 10) cases of IPA included [42, 43]. FDG-PET MRI has potential advantages over FDG-PET CT including lower total radiation dose; although there are currently no clinical data to define a role in diagnosis of IPA [39]. Whilst current FDG tracers are unable to differentiate between pathogens, novel *Aspergillus-*specific tracers in development have potential to improve specificity of PET-imaging for IPA in future clinical practice [44].

## Microbiological Diagnosis of IPA

Definitive diagnosis of IPA in children is challenging, due to the need for invasive testing, further compounded by the more frequent non-specific radiological appearances of fungal lung disease, compared to that seen in adults. Microbiological diagnosis not only allows timely administration of appropriate therapy in cases of IPA, but with increased use of mould-active prophylaxis in high-risk cohorts, the emergence of non-*Aspergillus* moulds in breakthrough IFD further necessitates accurate microbiological confirmation [45]. For IPA, there are fewer published data on non-culture-based biomarkers in children, and greater complexity in performing invasive tests, such as broncho-alveolar lavage (BAL) or lung biopsy [13, 15, 46]. Whilst microscopy, culture and or histopathological examination of direct respiratory samples remains the gold standard for diagnosis of IPA, the use of molecular methods and serum biomarkers have emerged as potentially useful additional tools (Table 2) (Supplementary Fig. 1). In cases where direct sampling is feasible, choice of sampling approach should consider the patient’s general condition, anticipated diagnostic yield, location of pulmonary lesions and potential complications [18]. According to revised consensus EORTC/MSG definitions, proven aspergillosis diagnosis in children requires invasive tests to meet mycological criteria, with recovery of *Aspergillus* sp. from a biopsy specimen [15]. Probable IFD can be established either by culture from BAL or sputum, or based on biomarkers from serum or BAL (Table 2) [15].

**Table 2:** Diagnostic microbiological tests for pulmonary aspergillosis: summary of recent guideline recommendations

Sample	Test	EORTC/MSG definition 2020 [15]	Guideline					Additional comments/data from systematic reviews
			ECIL 8 2021 [16]	ESCMID 2019 [13]	AAG 2021 [17]	IDSA 2016 [18]	IPFN 2017 [12]^^^^	
Bronchoalveolar lavage (BAL)	Microscopy & culture	Probable	*	*	A II	A II	–	Remains “gold-standard” for IPA diagnosis [17, 18]
	Galactomannan	Probable (ODI > 1.0)	A II (t)	B II (t)	A II^&^	A I	–	Sensitivity 82% Specificity 88% [61]
	*Aspergillus* PCR**	Probable	A II (t)^~^	N/A	B II	N/A	–	Pan-fungal PCR may be considered in high-risk patients [54]
Lung Biopsy	Histopathology & Culture	Proven	*	*	A II	A I	–	Remains “gold-standard” for IPA diagnosis [17, 18]
	*Aspergillus* or Panfungal PCR	(for species identification)	A II	N/A	A II^#^	N/A	–	Sensitivity > 90% Specificity 99% [17]^#^^^^
Serum (Serum/whole blood/plasma for PCR)	Galactomannan	Probable (ODI > 1.0)	A II^&^	B II	B II^&^	A I	C II^+^	Sensitivity 89% Specificity 85% [61]
	B-G-Glucan	N/A	D II	D III	C II^^^	A II^^^	D III	Sensitivity 29–82% Specificity 50–83% [61]^@^
	*Aspergillus* PCR	Probable	B II (t)	N/A	N/A	N/A	D II	Sensitivity 76% Specificity 58% [61]

AAG, Australasian Antifungal Guidelines; ECIL, European Conference on Infectious in Leukaemia; ESCMID, European Society for Clinical Microbiology and Infectious Diseases; IPFN, International Paediatric Fever and Neutropenia; ODI, optical density index; PCR, polymerase chain reaction

*Considered standard testing—appropriate efforts to identify causative pathogen recommended

^~^Recommendation for “PCR and molecular methods” whenever specimens are obtained

^#^Where fungal hyphae are visible (lower sensitivity for samples where fungal hyphae not visible—C II recommendation)

^+^Marginal recommendation against use in setting of prolonged fever and neutropenia

^^^General recommendation/data; no paediatric specific recommendation(s)/data provided

^&^ODI threshold of 0.5 recommended for clinical practice ^@^for proven/probable invasive fungal disease

**Two consecutive serum/whole blood/plasma samples positive OR BAL fluid 2 or more duplicate tests positive OR at least one plasma/serum/whole blood sample positive and one BAL fluid positive

^^^^This guideline is not specific for pulmonary aspergillus, but rather for children with suspected fungal infection in the setting of prolonged febrile neutropenia

Strength of recommendation: A, strong (dark green); B, moderate (light green); C, marginal (yellow); D, recommendation against use (lilac); N/A, no consensus recommendation made based on review of available data

Level of evidence: I—at least one properly designed randomised controlled trial/high quality; II—at least one properly designed clinical trial without randomisation, cohort or case-controlled studies, or multiple time series/moderate quality; III—evidence from opinions of respected authorities/low quality; (t) transferred evidence (i.e. from different patient cohorts, or similar immune status situation)

### Respiratory Sampling for IPA Diagnosis

Broncho-alveolar lavage, using a bronchoscope to instil normal saline into the lungs and reach distal airways, allows for recovery of alveolar fluid and pathogens. BAL has become a key diagnostic tool for invasive fungal and other types of pulmonary infections, in research and clinical practice [47]. Fluid obtained should be examined with cytology, as well gram and fluorescent staining, and fungal culture [17, 18, 48] (along with bacterial and viral testing). Microscopy, even with techniques such as fluorescent staining, is poorly sensitive for diagnosis of IPA (generally less than 50%) [48, 49] and diagnostic yield of fungi from paediatric BAL is reported in around 14% of procedures, compared with a proportion of 43% for all pathogens [50]. For peripheral lesions, BAL may be less sensitive than percutaneous biopsy [18]. Diagnostic yield for all pathogens in children has been reported to be superior with early BAL (< 72 h of symptoms) in one single-centre study [51] but no difference was found in another, though the latter study reported late BAL had higher utility in changing clinical management [52]. Mild adverse events are relatively common following BAL, reported in up to 19% of cases, but serious adverse events are uncommon [50].

Poor sensitivity of fungal microscopy and culture has led to development of additional tests for direct detection in these specimens. These include molecular assays such as PCR for specific pathogens, such as *Aspergillus* spp. *Aspergillus* PCR is typically much more sensitive than culture for diagnosis of IPA [17] and has now been included in the EORTC/MSG criteria as mycological evidence of disease [15]. *Aspergillus* PCR testing of all BAL samples is recommended in some recent guidelines (Table 2) [16, 17]. So-called ‘panfungal’ PCR, using targets from conserved fungal genetic sequences, which are subsequently sequenced for species identification (e.g. the internal transcribed spacers 1 and 2: ITS1 and ITS2 regions), may also be performed on BAL samples [53]. Panfungal PCR may also identify commensal organisms, however, and cost-effectiveness may require strategies to limit routine panfungal PCR testing of BAL fluid to high-risk patients [54].

Galactomannan (GM) a component of the *Aspergillus* cell wall, released during fungal growth, can be detected in BAL fluid and is more sensitive than fungal culture. GM is reported as an optical density index (ODI), with a threshold of ≥ 1.0 ODI in BAL fluid meeting criteria for probable disease [15]. False negatives are reported more often in those on mould-active antifungal prophylaxis and false positives due to bifidobacteria colonisation may occur, particularly in neonates and young children [46, 55, 56]. False positives have also previously been observed following administration of older formulations of piperacillin-tazobactam [56]. For children with suspected IPA, GM testing of BAL fluid, where available, is consistently recommended across recent consensus guidelines (Table 2) [13, 16–18].

Lung biopsy allows for all of the tests above, with the addition of histopathological examination of a sterile tissue sample [57], but is less commonly performed than BAL, due to higher complication rates (37% vs. 8% in children in one systematic review) [58]. This diagnostic procedure may be particularly considered when a non-infectious aetiology (e.g. malignant lesion) is also suspected, or when biopsy is considered for therapeutic intent [58, 59]. For biopsy specimens with histopathologically-confirmed, culture-negative invasive mould disease, panfungal PCR can assist in differentiating IPA from non-*Aspergillus* mould infections [15, 17].

### Non-invasive Diagnostic Tests for IPA

Considering the challenges with attaining invasive diagnostic samples, serum fungal biomarkers are a potentially attractive adjunct in the diagnosis of IPA. Non-invasive biomarkers including serum galactomannan (GM), serum B-D-glucan and *Aspergillus* PCR of blood, serum or plasma have been incorporated into adult clinical practice on the basis on data demonstrating clinical utility [17, 60]. Each of these assays has important limitations in children and there have been relatively few paediatric studies assessing performance [61]. In recent paediatric guidelines, of serum biomarkers, only GM is consistently recommended in the diagnosis of IPA (Table 2) [13, 16–18].

Serum Galactomannan can be used as a diagnostic test for children with suspected IPA [38] (use of serum GM as a screening test in children is beyond the scope of this review). A systematic review assessing serum biomarkers for IFD in paediatric cancer and HSCT included 8 studies assessing serum GM as a diagnostic test, in which prevalence of invasive aspergillosis ranged from 0 to 30.8% [61]. Negative predictive value for proven/probable aspergillosis was generally high (83–100%) but positive predictive value (4–100%) and specificity (39–100%) varied; pooled sensitivity was 89% and specificity 85% [61]. Considering test specificity, serum GM testing should be reserved for patients at high risk of IPA to limit false positive results. Importantly, sensitivity of GM is poorer in patients without underlying neutropenia, including in solid organ transplant recipients [17, 18, 60, 62] and for children with primary immunodeficiency (e.g. CGD and hyper IgE syndrome) [6, 18, 60, 63], limiting utility in these patients.

With increased uptake of anti-mould prophylaxis in children at high risk of IFD in accordance with recent guidelines [64], the ability of GM to detect IPA may be reduced, as demonstrated in adult patients [37, 61]. In the previous systematic review, no studies assessed GM in children receiving anti-mould prophylaxis [61]. Recently, analysis of data from a randomised trial of children and young adults with AML receiving antifungal prophylaxis with either caspofungin or fluconazole found very poor sensitivity (0%) of twice weekly GM surveillance testing in detecting cases of proven/probable IPA, although notably prevalence was < 2% in the overall cohort [65]. In children the performance of GM as a diagnostic test may also be limited by mould prophylaxis.

Despite significant limitations, the GM assay is still recommended as an adjunctive test in diagnosis of IPA in high-risk children [38, 62]. Although one previous international guideline recommended against use of GM in children with prolonged fever and neutropenia based on poor test specificity [12], other recent consensus guidelines recommend serum GM as part of initial workup in children with suspected IPA (Table 2) [13, 16, 17]. Notably, previous paediatric studies assessing GM as a diagnostic test have predominantly included children with clinical features such as neutropenia with or without fever, without consideration of concurrent diagnostic imaging results [61, 62]. The performance of serum GM in settings with higher pre-test probability, for example in high-risk children with consistent clinical and CT imaging features (see above), warrants further assessment, including in cohorts receiving anti-mould prophylaxis [62, 65].

B-D-Glucan is another component of the fungal cell wall which has been used as a biomarker of IFD. It is found in *Aspergillus* pp. but also several other fungi including *Candida* spp*., Fusarium* spp*., Scedosporium* spp. and *Pneumocystic jirovecii.* Previously, serum B-D-Glucan was included as part of the EORTC/MSG definition for probable IFD [20], but is no longer included in the updated definition of invasive mould disease [15]. The limited paediatric clinical data assessing serum B-D-Glucan as a diagnostic test generally indicate poor performance, and uncertainty remains as to the optimal test cut-off in children [38, 61, 66]. Based on available data, serum B-D-Glucan is not currently recommended in the diagnosis of IFD in children (Table 2) [13, 16, 67].

The performance of *Aspergillus* PCR testing of serum, whole blood or plasma, in limited available paediatric studies, has been variable [61]. Furthermore, standardised methods for PCR testing including sample processing, choice of primers and amplification have been lacking [38, 60, 61]. In the aforementioned systematic review, pooled sensitivity for *Aspergillus* PCR as a diagnostic test in 6 studies was 76% and specificity 58% [61]. Based on the available paediatric evidence, *Aspergillus* PCR testing of non-invasive samples in children with suspected IPA has not been recommended in previous guidelines [12, 13, 17, 18]. Of note, the updated EORTC/MSG criteria, positive *Aspergillus* PCR in blood is included in the definition of probable IPA [15], and the recent European Conference on Infections in Leukaemia guideline includes a recommendation for PCR of blood, serum or plasma in diagnosis of IPA in children (Table 2) [16].

### Emerging Microbiological Methods

There have been some promising developments in diagnostic methods with potential utility for children with suspected IPA in future. Matrix-assisted laser desorption time-of-flight mass spectrometry (MALDI-TOF MS), is readily available in many microbiology laboratories and with ongoing database improvements, is increasingly utilised to improve timeliness and accuracy of *Aspergillus* species identification of cultured clinical isolates [17]. *Aspergillus-*specific point-of-care lateral flow assays also have an emerging role in the diagnosis of IPA in adults, particularly in centres without ready access to GM assays, yet there are limited data in children to date [17]. Direct metagenomic next-generation sequencing has also shown promise in detection of fungal and non-fungal pathogens from BAL specimens, with added potential to rapidly detect genes associated with phenotypic resistance [68]. Elsewhere, next-generation sequencing of cell-free DNA from peripheral blood samples, referred to as “liquid biopsy” has demonstrated high specificity (95%) in diagnosis of invasive mould disease in adult patients, although sensitivity for pulmonary aspergillosis (31%) was modest [69]. As novel modalities emerge, efforts to standardise testing methods, develop clinical algorithms to guide use of existing and emerging tests, undertake multi-centre evaluation of performance in paediatric patients and advocate for registration in paediatric populations is required.

## Summary

For investigation of suspected IPA in immunocompromised children, CT imaging remains the modality of choice. Nodules with or without a surrounding halo are the most common finding and should prompt further investigation and consideration of pre-emptive treatment. Notably, no single imaging finding or combination of findings can be considered diagnostic of IPA, and pursuit of microbiological confirmation is vital, particularly in high-risk patients. With recent and ongoing changes to immunosuppressive and supportive care regimens along with improvements in imaging technology, ongoing evaluation of the diagnostic performance, overall and in specific subpopulations with suspected IPA is required to inform future diagnostic guidelines.

Direct respiratory sampling should be performed in all patients with suspected IPA, unless specific contraindications exist, to confirm the diagnosis and to guide antifungal therapy. Direct microbiological confirmation is particularly important with a relative increase in non-*Aspergillus* mould infections in the setting of more widespread use of prophylaxis targeting IPA. Non-invasive serum biomarkers, particularly serum GM are a useful adjunct in diagnosis in high-risk children with suspected IPA, but an understanding of test limitations is essential in interpreting results. Ongoing studies to optimise the use of available diagnostic tests in children with suspected IPA, as well as the concurrent development of new reliable microbiological modalities are priorities.

### Supplementary Information

Below is the link to the electronic supplementary material.

Supplementary file1 (TIF 451 KB)
