# Supplementary figures and images for: The Challenge of Diagnosing Invasive Pulmonary Aspergillosis in Children: A Review of Existing and Emerging Tools
Source: Mycopathologia. 2023 Apr 8;188(5):731–43. doi: 10.1007/s11046-023-00714-4 (PMC10564821; doi:10.1007/s11046-023-00714-4)

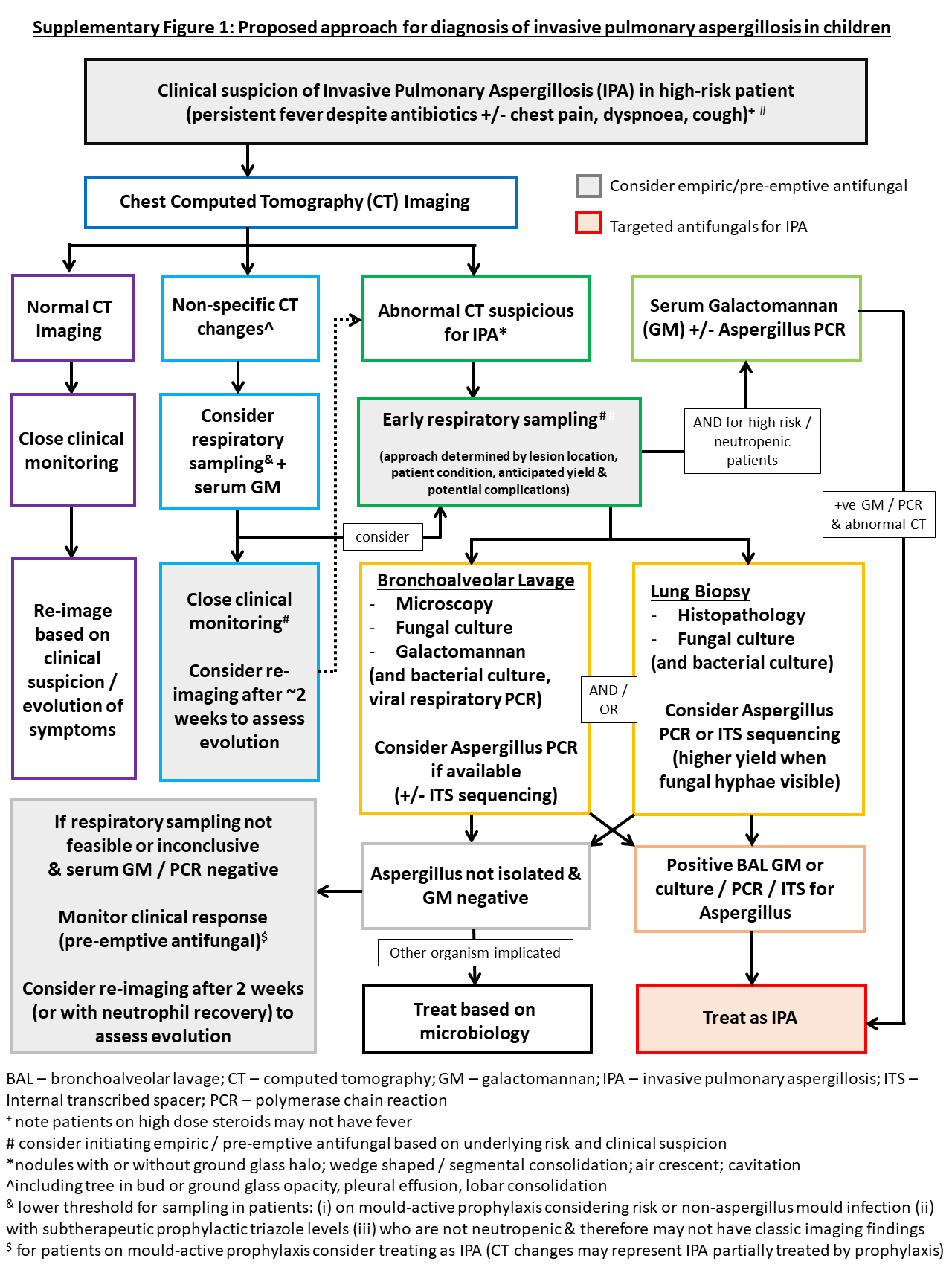

Supplement: Supplementary file 1 — Supplementary file1 (TIF 451 KB) [file 11046_2023_714_MOESM1_ESM.tif]
